# Philadelphia School of Examinations

**Published:** 1868-04-01

**Authors:** 


### *Philadelphia School of Examinations.*

We notice that our Philadelphia correspondent, Dr. Hutchins, in connection with Drs. Allis and Townshend, has opened in that city a course of examinations upon the lectures delivered in the Jefferson College, commencing April 2nd ensuing. $30 per course. Office students (including examinations), $100. This is one of the practical methods of "elevating the profession." The gentlemen connected with the Spring Course of Rush Medical College are undertaking a similar work. The college sessions are long enough — let the interim every where be filled up by daily recitations and clinical teaching.

